# Combined analysis of mRNA expression of ERCC1, BAG-1, BRCA1, RRM1 and TUBB3 to predict prognosis in patients with non-small cell lung cancer who received adjuvant chemotherapy

**DOI:** 10.1186/1756-9966-31-25

**Published:** 2012-03-23

**Authors:** Xue-Feng Leng, Ming-Wu Chen, Lei Xian, Lei Dai, Guang-Yao Ma, Man-Hong Li

**Affiliations:** 1 Department of Cardiothoracic Surgery, The First Afflicted Hospital of Guangxi Medical University, 22# Shuangyong Road, Qingxiu Region 530021 Nanning, China; 2 Department of Cardiothoracic Surgery, The First Afflicted Hospital of Luohe Medical college, 462000 Luohe, China; 3 Department of Cardiothoracic Surgery, Central Hospital of Loudi, 417000 Loudi, China

**Keywords:** Non-small cell lung cancer, ERCC1, BAG-1, BRCA1, RRM1, TUBB3

## Abstract

**Background:**

The aim of this study was to investigate prognostic value of excision repair cross-complementing 1 (ERCC1), BCL2-associated athanogene (BAG-1), the breast and ovarian cancer susceptibility gene 1 (BRCA1), ribonucleotide reductase subunit M1 (RRM1) and class III β-tubulin (TUBB3) in patients with non-small cell lung cancer (NSCLC) who received platinum- based adjuvant chemotherapy.

**Methods:**

Messenger RNA expressions of these genes were examined in 85 tumor tissues and 34 adjacent tissue samples using semi-quantitative RT-PCR. The expressions of these five genes were analyzed in relation to chemotherapy and progression-free survival (PFS) and overall survival (OS). Seventy-four patients were enrolled into chemotherapy.

**Results:**

Patients with ERCC1 or BAG-1 negative expression had a significantly longer PFS (*P *= 0.001 and *P *= 0.001) and OS (*P *= 0.001 and *P *= 0.001) than those with positive expression. Patients with negative ERCC1 and BAG-1 expression benefited more from platinum regimen (*P *= 0.001 and *P *= 0.002). Patients with BRCA1 negative expression might have a longer OS (*P *= 0.052), but not PFS (*P *= 0.088) than those with BRCA1 positive expression. A significant relationship was observed between the mRNA expression of ERCC1 and BAG-1 (*P *= 0.042). In multivariate analysis, ERCC1 and BAG-1 were significantly favorable factors for PFS (*P *= 0.018 and *P *= 0.017) and OS (*P *= 0.027 and *P *= 0.022).

**Conclusions:**

ERCC1 and BAG-1 are determinants of survival after surgical treatment of NSCLC, and its mRNA expression in tumor tissues could be used to predict the prognosis of NSCLC treated by platinum.

## Background

Lung cancer is a common malignant tumor, and was the first ranked cause of cancer death in both males and females [1]. As one of the most prevalent malignant tumors in China, lung cancer has been highlighted with emphasis for cancer prevention and treatment. Recently, the combinations of cytotoxic agents (such as gemcitabine, vinorelbine, and taxane) and platinum become new standard for non-small-cell lung cancer (NSCLC). But the resistance to these drugs causes unsatisfactory of overall survival rate. Therefore, it is very important to understand the molecular markers of resistance to chemotherapeutic drugs.

The excision repair cross-complementing 1 (ERCC1) is a DNA damage repair gene that encodes the 5' endonuclease of the NER complex, and is one of the key enzymes of the nucleotide excision repair (NER) pathway which is essential for the removal of platinum-DNA adducts. Clinical studies have found that high ERCC1 expression is associated with resistance to platinum-based chemotherapy and worse prognosis in patients with advanced NSCLC [2]. The human BAG-1 gene is located in chromosome 9 and encodes three major BAG-1 isoforms, BAG-1S (p36), BAG-1 M (p46), and BAG-1 L (p50), which are generated via alternate translation mechanisms from the same mRNA [3]. BAG-1 is a multifunctional binding protein involved in differentiation, cell cycle, and apoptosis. BAG-1 has recently been found to bind and interact with the anti-apoptotic gene Bcl-2, thereby inhibiting apoptosis [4]. Because of its affect on apoptosis, BAG-1 may play an important role in lung cancer. Further study showed that BAG-1 could be a target for lung cancer treatment of cisplatin [5]. The breast and ovarian cancer susceptibility gene1 (BRCA1) was the first breast cancer susceptibility gene identified in 1990 and was primary cloned in 1994. It has multiple roles not only in DNA damage repair but also in cell cycle regulation, transcriptional control, ubiquitination and apoptosis. In NSCLC, chemotherapeutic treatment can damage DNA through various mechanisms, the lack of functional BRCA1 can lead to increased sensitivity of the tumor cells to molecular damage, demonstrating that BRCA1 represents a predictive marker of chemotherapy response in NSCLC [6].

Ribonucleotide reductase subunit M1 (RRM1) is located on chromosome segment 11p15.5, it is a region with a frequent loss of heterozygosity in NSCLC. It is a component of ribonucleotide reductase, which is required for deoxynucleotide production and is also the predominant cellular determinant of the efficacy of gemcitabine, which make it to be the molecular target of gemcitabine [7,8]. Along with the use of antitubulin agents such as taxanes and vinorelbine, study shows there are a number of tubulin isotypes in humans, and found that class III β-tubulin (TUBB3) among them is expressed in a proportion and related to clinical outcome [9]. The expression of TUBB3 is associated with resistance of paclitaxel and docetaxel, no matter in vitro or in clinical research [10,11].

Changes of gene mRNA expression during carcinogenesis may lead impact of the diagnosis, treatment, and prevention of NSCLC, it is important to understand these changes. So, in this study, we use RT-PCR to examine the expression of ERCC1, BAG-1, BRCA1, RRM1 and TUBB3 in tumor samples from patients with resected NSCLC not receiving adjuvant chemotherapy. We analyzed the relationships of these genes expression in tumors about survival time and response to chemotherapy to determine whether the expression of these molecules could be used as prognostic factors of progression-free and overall survival in this cohort of patients.

## Methods

### Patient data

A total of 85 patients who underwent curative surgery for NSCLC between August 2007 and April 2009 were enrolled into this study, including 85 tumor tissues and 34 adjacent tissues respectively. Among them there were 60 males and 25 females, aged 24-84 (mean 57) years. According to WHO Classification (2000), there were 25 squamous, 60 adenomatous, with 58 moderate and well differentiated (G1-G2) and 27 low differentiated (G3). Because there were only 4 cases of stage IV patients who all had surgery after single brain metastasis resected firstly, and there were no patients of stage IIIb. On account of stage IV patients were too few, so we combined 48 cases as staged I-II and 37 III-IV based on the revised AJC staging for lung cancer (1997). 28 cases had intra-thoracic lymph node metastasis (N1-N2), and 57 were negative lymph node metastasis. Additional information of surgery and chemotherapy status were all showed in (Table 1). The paracancerous tissues (defined as more than 5 cm away from the carcinoma tissue) taken from 34 cases were used as controls. A total of 74 Patients received at least two cycles of adjuvant chemotherapy within three months after surgery in the First Afflicted Hospital of Guangxi Medical University. 34 of 74 patients were received GP (Cisplatin 75 mg/m^2 ^on day 1, Gemcitabine 1000 mg/m^2 ^on days 1,8), 29 of 74 patients were received NP (Cisplatin 75 mg/m^2 ^on day 1, Vinorelbine 25 mg/m^2 ^on days 1 + 8), the other 11 patients were received TP (Carboplatin AUC 6 on day 1, Paclitaxel 175 mg/m^2 ^on day 1), every 3 weeks. All of the tumor tissue samples were freshly frozen in liquid nitrogen immediately after surgery, and stored at -80 ^0 ^C until analysis was available. We took out the specimens from the parenchymal tissues of tumor, and we must as far as possible make the specimens keep away from the necrotic tissue. We also confirmed the HE stain results from the pathology department after surgery, which tumor sections, from the location specimens taken by us, were full of tumor cells (usually more than 60%-70%). Patients who received neoadjuvant chemotherapy or neoadjuvant radiotherapy were excluded. The study protocol was approved by the Ethical Committee of the First Affiliated Hospital of Guangxi Medical University, China. All subjects signed an informed consent before entry into the study.

**Table 1:** Baseline characteristics of 85 patients with NSCLC

Characteristics	Number	Percentage (%)
**Gender**		
Male	60	70.6
Female	25	29.4

**Age**		
≤ 60	53	62.4
> 60	32	37.6

**Nationality**		
The Han	60	70.6
The Zhuang	25	29.4

**Histology**		
Squamous carcinoma	25	29.4
Adenocarcinoma	60	70.6

**Differentiation**		
Well and moderate	58	68.2
Poor	27	31.8

**Metastasis lymphatics**		
Yes	28	32.9
No	57	67.1

**TNM stage**		
I + II	48	56.5
III + IV	37	43.5

**Surgery status**		
Lobectomy	79	92.9
Pneumonectomy	6	7.1

**Chemotherapy status**(74 cases)		
GP regimens	34	45.9
NP regimens	29	39.2
TP regimens	11	14.9

**ECOG Performance status**		
0	22	25.9
1	63	74.1

### RNA isolation and cDNA synthesis

Fresh frozen specimens of tumor and adjacent tissues were obtained from 85 patients. Collection time from resection to freezing was required 20 minutes or less for all specimens. The fresh frozen specimens were processed for RNA isolation and reverse-transcriptase polymerase chain reaction (RT-PCR) in detecting expression analysis for the ERCC1, BAG-1, BRCA1, RRM1, and TUBB3 genes. Specimens were microscopically examined to assess quality and to verify the histopathology.

**Figure 1:**
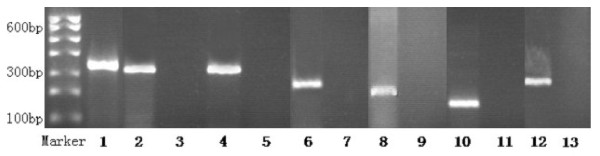
**The expression of ERCC1, BAG-1, BRCA1, RRM1 and TUBB3 in NSCLC tissues**. 1: β-actin; 2: positive control of ERCC1; 3: negative control; 4-5: positive and negative expression of ERCC1; 6-7: positive and negative expression of BAG-1; 8-9: positive and negative expression of BRCA1; 10-11: positive and negative expression of RRM1; 12-13: positive and negative expression of TUBB3.

Specimens were pulverized by pulp refiner under Trizol reagent (Invitrogen). Total RNA was extracted with Trizol reagent and dissolved in DEPC water. Total RNA were reverse transcribed with RevertAid™ First Strand cDNA Synthesis Kit (Fermentas) for generation of cDNA. Gene expression for ERCC1, BAG-1, BRCA1, TUBB3, RRM1 and β-actin (internal reference gene) were performed using RT-PCR. The preliminary experiment and large sample experiment PCR were carried out as follows: an initial denaturation at 94°C for 3 min 30 s, followed by 30 cycles of denaturation at 94°C for 40 s, annealing at different temperature for different gene for 40 s, and elongation at 72°C for 50 s, then elongation 72°C for 7 min at last. The sequences of the primers used were in Table 2. All of these primers were checked and met a high specificity by BLAST function in NCBI. Confirmative PCR products through gene sequencing were used as positive controls to exclude false negative, and the no template added reaction system used as negative controls to exclude contamination of genomic DNA (Figure 1).

**Table 2:** Primers for gene analysis

Gene	Accession Number	Primer sequence(5'-3')	Product length	Tm
ERCC1	NM_001983.3	Forward 5'-CCCTGGGAATTTGGCGACGTAA-3'	273 bp	59°C

		Reverse 5'-CTCCAGGTACCGCCCAGCTTCC-3'		

BAG1	NM_004323.5	Forward 5'-GGCAGCAGTGAACCAGTTG-3'	242 bp	54.5°C

		Reverse 5'-GCTATCTTCTCCACAGACTTCTC-3'		

BRCA1	NM_007294.3	Forward 5'-AAGGTTGTTGATGTGGAGGAG-3'	208 bp	55.6°C

		Reverse 5'-CAGAGGTTGAAGATGGTATGTTG-3'		

RRM1	NM_001033.3	Forward 5'-TGGCCTTGTACCGATGCTG-3'	161 bp	57.5°C

		Reverse 5'-GCTGCTCTTCCTTTCCTGTGTT-3'		

TUBB3	NM_006086.3	Forward 5'-CGGATCAGCGTCTACTAC-3'	222 bp	49°C

		Reverse 5'-CACATCCAGGACCGAATC-3'		

β-actin	NM_001101.3	Forward 5'-CTCGCGTACTCTCTCTTTCTGG-3'	334 bp	60°C

		Reverse 5'-GCTTACATGTCTCGATCCCACTTAA-3'		

### Statistical analysis

The data were analyzed using SPSS 17.0 software package. The correlation of gene expression with different clinical characteristics was analyzed with chi-square test or Fisher's exact test. Correlation between gene mRNA levels was evaluated by Spearman correlation coefficients. The Kaplan-Meier method and Log-rank test were used to analyze the correlation of patient survival with gene expression. Factors with significant influence on survival in univariate analysis were further analyzed by multivariate Cox regression analysis. A significance level of *P *< 0.05 was used.

## Results

### Expression of ERCC1, BAG-1, BRCA1, RRM1 and TUBB3 mRNA after surgical resection

**Figure 2:**
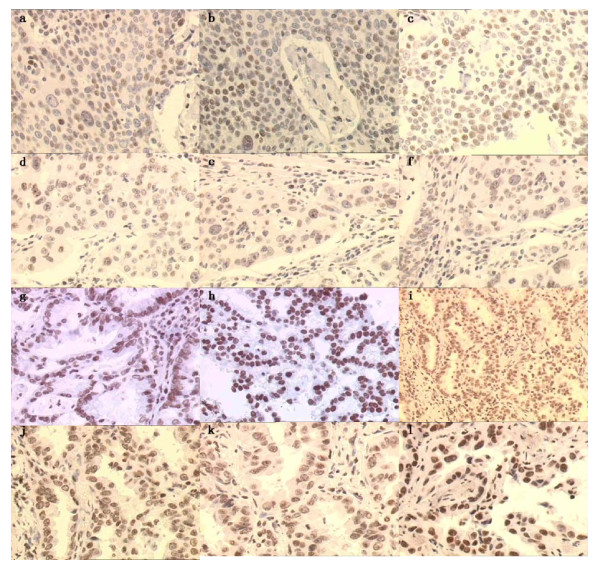
**Immunohistochemical staining of ERCC1 proteins in NLCLC tissues**. Expression of ERCC1 protein was detected in the nuclei of cancer cells. a-f: squamous carcinoma; g-l: adenocarcinoma.

Tumor specimens from 85 patients were available for the analysis of these genes mRNA. The specimens included 85 tumor tissues and 34 adjacent tissues. The positive rate of ERCC1 mRNA in tumor and its adjacent tissues were 58.8% and 55.9% respectively (*P *= 0.769). BAG-1 were 37.6% and 82.4% (*P *= 0.000). BRCA1 were 16.5% and 44.1% (*P *= 0.002). RRM1 were 30.8% and 38.2% (*P *= 0.105). TUBB3 were 16.5% and 2.9% (*P *= 0.089). We chose some of the same samples which ERCC1 mRNA expressions were positive in order to validate the results. Expression of ERCC1 proteins was assessed by immunohistochemistry, and expression of the ERCC1 proteins was detected in the nuclei of cancer cells. All the samples selected were ERCC1 positive, including 6 squamous carcinoma and 6 adenocarcinoma (Figure 2).

### Correlation between ERCC1, BAG-1, BRCA1, RRM1 and TUBB3 expression and clinical features

The expression of five genes in different clinical features were compared and summarized. It showed that the difference of these five genes were only significant between some parts of clinical features. Correlations were observed between ERCC1 expression and TNM stage (*P *= 0.006), metastasis of lymph node (*P *= 0.01), and TUBB3 expression and TNM stage (*P *= 0.004). No Correlation was observed between ERCC1, TUBB3 expression and other clinical features. Besides, No Correlation was observed between BAG-1, BRCA1, RRM1 expression and gender, age, nationality, histology, differentiation of tumor, metastasis of lymph node, TNM stage, chemotherapy status or performance status.

### Association between gene expression and survival after surgical resection

**Figure 3:**
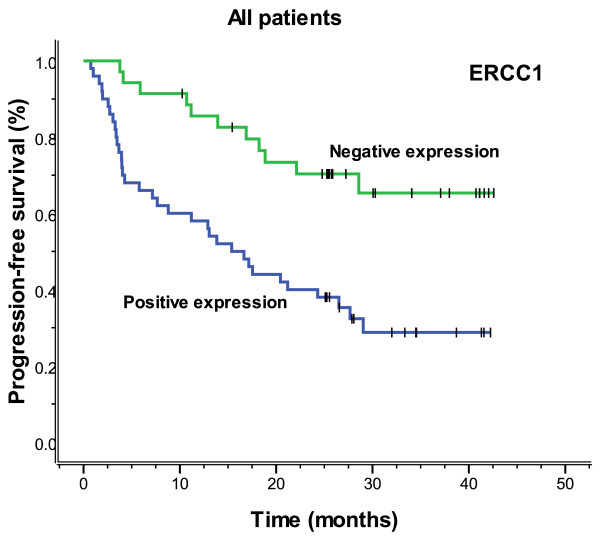
**Progression-free survival according to ERCC1 expression (more than 42.6 vs. 15.4 months, *P *= 0.001)**.

**Figure 4:**
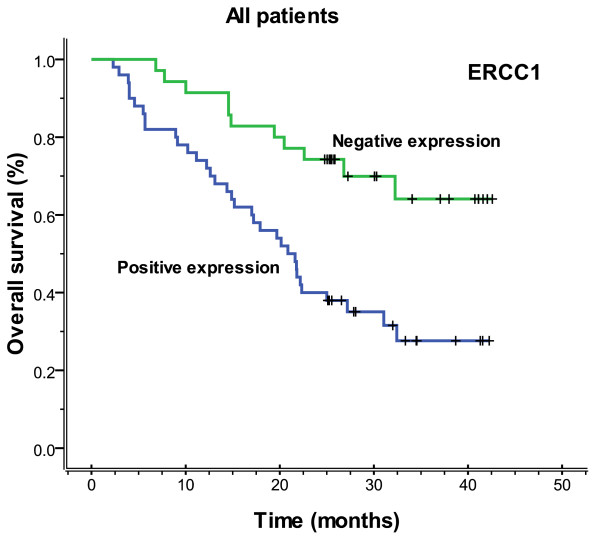
**Overall survival according to ERCC1 expression (more than 42.6 vs. 20.9 months, *P *= 0.001)**.

**Figure 5:**
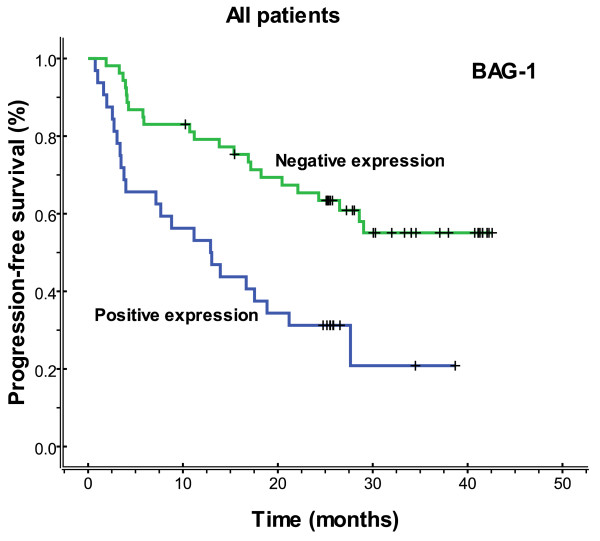
**Progression-free survival according to BAG-1 expression (more than 42.6 vs. 12.9 months, *P *= 0.001)**.

**Figure 6:**
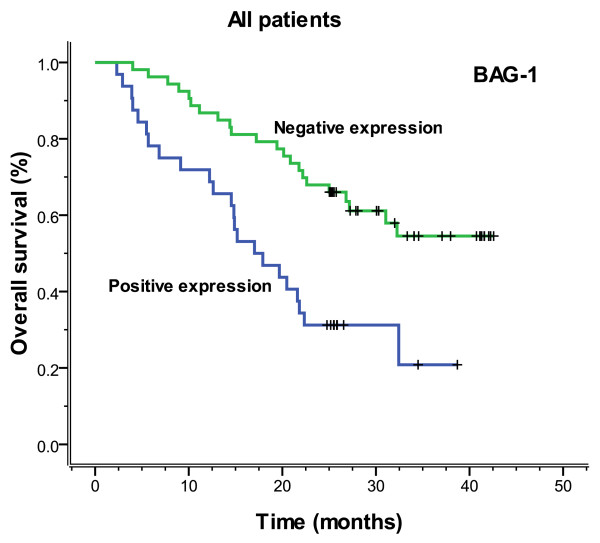
**Overall survival according to BAG-1 expression (more than 42.6 vs. 17.0 months, *P *= 0.001)**.

The median follow-up time was 23.3 months (range 2.3-42.6), and the median overall survival and median PFS (progression-free survival) were 27.2 months (range 2.3-42.6) and 26.5 months (range 0.8-42.6), respectively. Figures 3, 4, 5 and 6 showed the Kaplan-Meier survival curves in patients positive and negative for ERCC1 and BAG-1 expression. Patients negative for ERCC1 expression had a significantly longer median progression-free (more than 42.6 vs. 15.4 months. *P *= 0.001) and overall (more than 42.6 vs. 20.9 months. *P *= 0.001) survival, compared with those positive for ERCC1 expression. Patients negative for BAG-1 expression had a significantly longer median progression-free survival (more than 42.6 vs. 12.9 months. *P *= 0.001) and overall survival (more than 42.6 vs. 17.0 months. *P *= 0.001), than those positive for BAG-1 expression. The relationships between the PFS and BRCA1, RRM1 and TUBB3 were no statistical significance (*P *= 0.088, *P *= 0.116 and *P *= 0.271), and there were also the same results for OS (*P *= 0.057, *P *= 0.110 and *P *= 0.342).

Median value of clinicopathologic factors and expression of genes of tumor samples were used as a cut-off point at univariate analysis. Univariate Cox analysis was carried out to identify the factors that were significantly associated with progression-free and overall survival (Table 3). In the univariate analysis, ERCC1 expression (*P *= 0.001), BAG-1 expression (*P *= 0.001), TNM stage (*P *= 0.007) and metastasis of lymph node (*P *= 0.006) were prognostic of progression-free survival. For overall survival, ERCC1 expression (*P *= 0.002), BAG-1 expression (*P *= 0.001), TNM stage (*P *= 0.008) and metastasis of lymph node (*P *= 0.007) were also prognostic. None of the other factors examined were statistically correlated with progression-free or overall survival for ERCC1, BAG-1 and the other three genes.

**Table 3:** Univariate analysis of Clinicopathological features, tumor markers, and patient survival

Variable	PFS HR (95% CI)	*P *value	OS HR (95% CI)	*P *value
Gender (Male vs. Female)	1.370 (0.744-2.524)	0.313	1.341 (0.713-2.421)	0.381

Age (≤ 60 vs.>60)	1.433 (0.789-2.604)	0.237	1.450 (0.798-2.635)	0.223

Nationality (The Han vs. The Zhuang)	0.929 (0.480-1.800)	0.827	0.964 (0.497-1.867)	0.912

Histology (Squamous carcinoma vs. Adenocarcinoma)	0.541 (0.267-1.095)	0.088	0.559 (0.276-1.133)	0.106

Differentiation (Well and moderate vs. Poor)	0.992 (0.528-1.866)	0.980	0.953 (0.506-1.795)	0.881

Metastasis lymphatics (Yes vs. No)	0.429 (0.236-0.780)	0.006**	0.435 (0.238-0.793)	0.007**

TNM stage (I+II vs. III+IV)	2.267 (1.257-4.090)	0.007**	2.217 (1.227-4.003)	0.008**

ERCC1 (positive vs. negative)	0.326 (0.165-0.645)	0.001**	0.333 (0.169-0.660)	0.002**

BAG-1 (positive vs. negative)	0.367 (0.202-0.665)	0.001**	0.363 (0.200-0.658)	0.001**

BRCA1 (positive vs. negative)	0.546 (0.270-1.105)	0.093	0.505 (0.250-1.021)	0.057

RRM1 (positive vs. negative)	0.539 (0.314-1.143)	0.120	0.590 (0.309-1.126)	0.110

TUBB3 (positive vs. negative)	0.665 (0.319-1.383)	0.275	0.701 (0.338-1.458)	0.342

** represent *P *< 0.01

Multivariate Cox regression analysis was performed to evaluate the influence of these genes on the progression-free survival adjusting for possible confounding factors. From the results of the univariate analysis, TNM stage and metastasis of lymph node, also ERCC1 and BAG-1 were significantly correlated to the progression-free survival (Table 4). After multivariate analysis, ERCC1 was statistically significant (*P *= 0.018) and the hazard ratio was 0.0427 (95% CI: 0.211-0.864). BAG-1 was also statistically significant (*P *= 0.017) and the hazard ratio was 0.0474 (95% CI: 0.257-0.874). However, the *P*-value for TNM stage (*P *= 0.340, 95% CI: 0.336-1.457) and lymph node (*P *= 0.217, 95% CI: 0.299-1.315) were not statistically significant.

**Table 4:** Multivariate analysis of Clinicopathological features, tumor markers, and patient survival

Variable	PFS HR (95% CI)	*P *value	OS HR (95% CI)	*P *value
ERCC1 (positive vs. negative)	0.427 (0.211-0.864)	0.018*	0.447 (0.219-0.911)	0.027*
BAG-1 (positive vs. negative)	0.474 (0.257-0.874)	0.017*	0.486 (0.262-0.901)	0.022*
Metastasis lymphatics (Yes vs. No)	0.627 (0.299-1.315)	0.217	0.654 (0.352-1.370)	0.260
TNM stage (I + II vs. III + IV)	0.699 (0.336-1.457)	0.340	1.442 (0.691-2.984)	0.324

* represent *P *< 0.05

Multivariate Cox regression analysis was also performed for the overall survival. In addition to ERCC1, BAG-1, TNM stage and metastasis of lymph node were included in the Cox models. The other gene of TUBB3, BRCA1 and RRM1, together with the rest of patients' and clinical characteristics were not included. Negative ERCC1 and BAG-1 expression were independent and significant predictor of favorable outcome for overall survival (*P *= 0.027 and *P *= 0.022), with a hazard ratio of ERCC1 was 0.447 (95% CI: 0.219-0.911); for BAG-1, with a hazard ratio of 0.486 (95% CI: 0.262-0.901), whereas TNM stage and metastasis of lymph node had no significant association. The reason that TNM staging and lymph node were not associated with survival in the multivariate analysis might be the statistical significance of the two characteristics with survival contained in the other variables (ERCC1 and BAG-1). The other explanatory reason might be the limit of sample size.

### Correlations between ERCC1, BAG-1, BRCA1, RRM1 and TUBB3 expression and the kind of adjuvant chemotherapy

74 of 85 patients received at least two cycles of adjuvant chemotherapy, of whom 66 (89.2%) finished at least 4 cycles. The main chemotherapy regimens included gemcitabine (GEM, 45.9%), vinorelbine (NVB, 39.2%) and paclitaxel (PTX, 14.9%) combined with cisplatin (DDP)/carboplatin (CBP).

**Figure 7:**
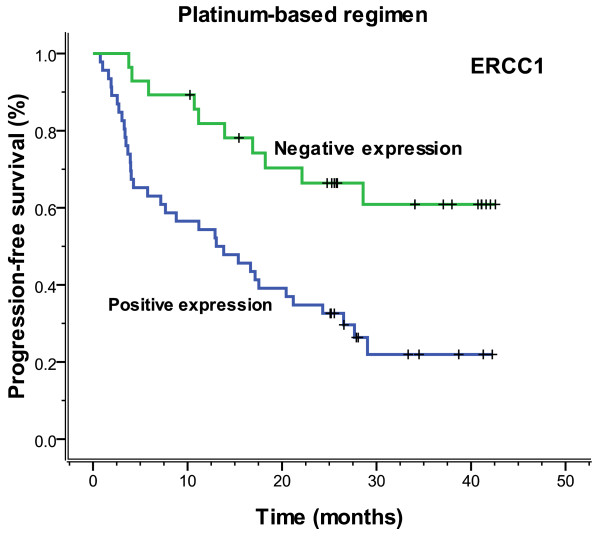
**Progression-free survival according to ERCC1 expression which was based on platinum chemotherapy (more than 42.6 vs. 13.0 months, *P *= 0.001)**.

**Figure 8:**
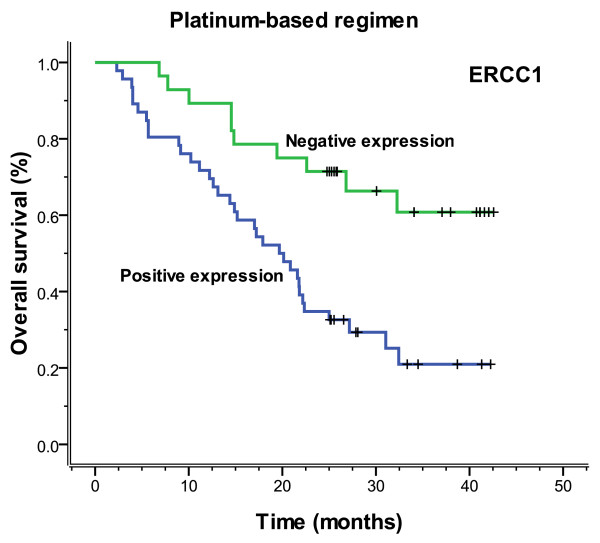
**Overall survival according to ERCC1 expression which was based on platinum chemotherapy (more than 42.6 vs. 19.7 months, *P *= 0.001)**.

**Figure 9:**
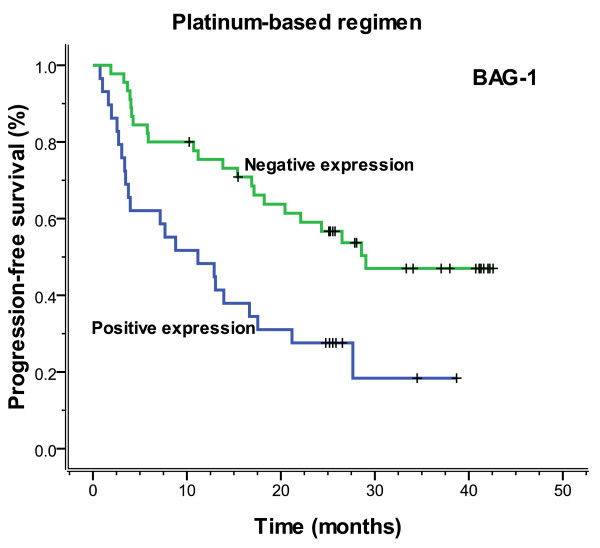
**Progression-free survival according to BAG-1 expression which was based on platinum chemotherapy (29.0 vs. 11.2 months, *P *= 0.002)**.

**Figure 10:**
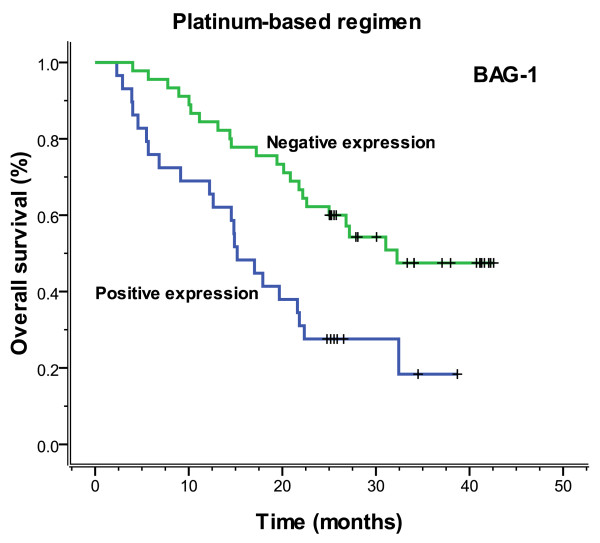
**Overall survival according to BAG-1 expression which was based on platinum chemotherapy (32.3 vs. 15.2 months, *P *= 0.002)**.

In 74 patients treated with the regimen of cisplatin/carboplatin, patients negative for ERCC1 expression had a significantly longer median progression-free (more than 42.6 months vs. 13.0 months, *P *= 0.001) and overall (more than 42.6 months vs. 19.7 months, *P *= 0.001) survival, compared with those positive for ERCC1 expression (Figures 7, 8). Patients negative for BAG-1 expression also had a significantly longer median progression-free survival (29.0 months vs. 11.2 months, *P *= 0.002) and overall survival (32.3 months vs. 15.2 months, *P *= 0.002), than those positive for BAG-1 expression (Figures 9, 10). Whereas, there was no statistical significance in progression-free and overall survival to patients with BRCA1 expression (*P *= 0.129 and *P *= 0.073, respectively). In those treated with the regimen of gemcitabine, there was no statistical significance found in progression-free and overall survival for patients with RRM1 expression (*P *= 0.310 and *P *= 0.299, respectively). In the anti-tubulin regimen group of vinorelbine or paclitaxel, no statistical significance was found in progression-free and overall survival between the negative and positive expression of TUBB3 (*P *= 0.745 and *P *= 0.742, respectively); in the same measure, no statistical significance was found in progression-free and overall survival between the negative and positive expression of BRCA1 (*P *= 0.612 and *P *= 0.389, respectively).

### Correlation of ERCC1 and BAG-1 expression

**Figure 11:**
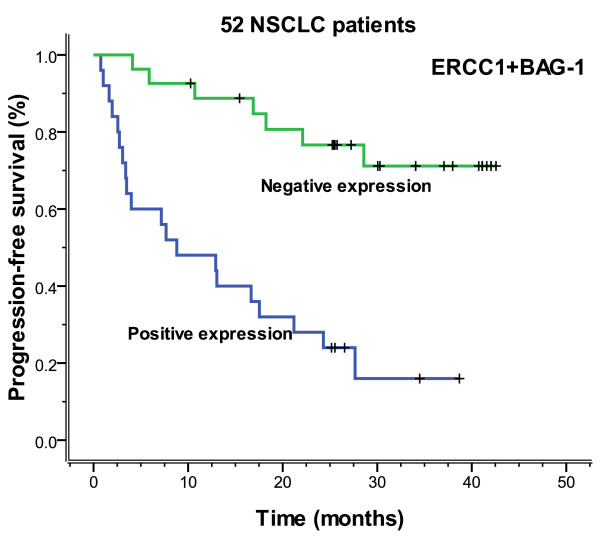
**Progression-free survival according to 52 NSCLC patients who have both ERCC1 and BAG-1 expression, all of whom were based on platinum chemotherapy (more than 42.6 vs. 8.8 months, *P *= 0.000)**.

**Figure 12:**
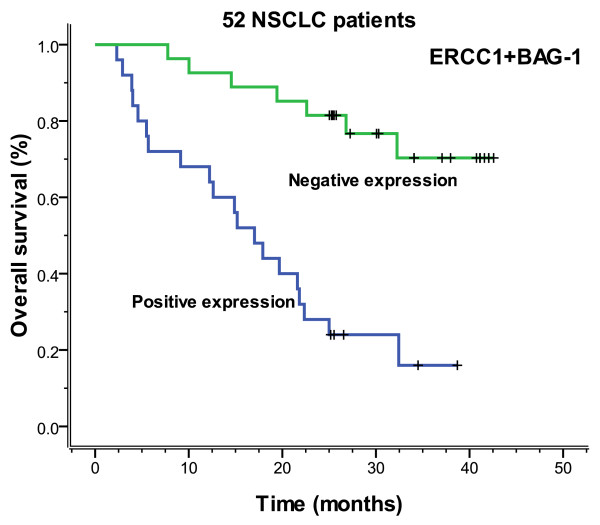
**Overall survival according to 52 NSCLC patients who have both ERCC1 and BAG-1 expression, all of whom were based on platinum chemotherapy (more than 42.6 vs. 17.0 months, *P *= 0.000)**.

There were 25 cases that expressed both ERCC1 and BAG-1 and 27 cases that expressed neither. As shown in Table 5, the correlation was found between ERCC1 and BAG-1 gene expression (*P *= 0.042, r = 0.247). All 52 patients of both positive and negative expression were received adjuvant chemotherapy. For both negative mRNA expression had a significantly longer median progression-free (more than 42.6 months vs. 8.8 months, *P *= 0.000) and overall (more than 42.6 months vs. 17.0 months, *P *= 0.000) survival, compared with those positive for both ERCC1 and BAG-1 expression (Figures 11, 12).

**Table 5:** Correlation between expression of ERCC1 and BAG-1

Gene			ERCC1	
		+		-

	+	25		8

BAG-1				

	-	25		27

## Discussion

Along with the development of theory and practice in treatment of chemotherapy with resected NSCLC, we have already known the combination of two cytotoxic drugs, like a platinum and a non-platinum agent, is the standard first-line treatment of NSCLC patients [12]. However, because of the high rate of toxicity observed and associated with drug resistance, treatment response rate and median overall survival are not satisfactory. This appears to be gene of chemoresistance, which plays an important role in the after surgery treatment. So, some markers detection is a key for chemotherapy in NSCLC patients.

Platinum drugs mainly exert their cytotoxicity by forming bulky intra-strand platinum-DNA adducts and inter-strand cross-link of the two DNA strands. Removal of these adducts from genomic DNA and repair of inter-strand cross-links in DNA and recombination processes are mediated by components of different DNA repair pathways. ERCC1 is a key factor involved in nuclear excision repair (NER) for platinum induced adducts [13]. There is observation of platinum resistance in lung cancer A549 cells lines with high expression of ERCC1 [14], and increased clinical evidence that overexpression of ERCC1 in NSCLC inhibits platinum efficacy. In addition to ERCC1 negative tumors appear to benefit from cisplatin based chemotherapy, it also gains benefit from overall survival as a prognostic factor [2,15,16]. As a predictive factor, a phase III trial in NSCLC showed better PFS and OS in the low genotypic than in the high genotypic group, and the patients in the low genotypic group also had a trend toward a lower risk of progression than those in the control arm [17]. Although it had been identified that ERCC1 positive could be associated with resistance to platinum based chemotherapy, however, some studies reported that ERCC1 expression had correlation with improved prognosis [18,19]. This discrepancy may be due to differences of experimental processing, regional disparity or technical issues. In our study, expression of ERCC1 in stage III + IV was higher than stage I + II (*P *= 0.006). This was also happened in lymph node metastasis compared to no metastasis (*P *= 0.01), which like Ota et al. reported [20]. The available data indicate ERCC1 positive patients might present a poor prognosis, and ERCC1 expression might appear to be an advanced stage event.

The BAG-1, as an anti-apoptotic function, exhibits positive expression in many malignant tumors. It binds to the cytosolic domain of the growth factor receptors on the cell surface, enhancing the protection from cell death triggered by these receptors. However, it binds to Bcl-2 and heat shock protein (HSP) and modulates their function in the cytosol, and it binds to nuclear hormone receptors for inhibiting hormone-induced apoptosis in the nucleus [21]. Further exploration shows overexpression of BAG-1 suppresses activation of caspases and apoptosis induced by chemotherapeutic agents [22]. As expected, experiment performed in lung cancer cells indicates silencing of BAG-1 gene can sensitize lung cancer cells to cisplatin-induced apoptosis [5]. In this study, the positive BAG-1 expression correlated significantly with progression-free and overall survival in patients treated by platinum. As we described, current research has proven expression of BAG-1 indicates poor prognosis [23]. Whereas, Rorke et al. [24] reported high expression of BAG-1 may correlate to better prognosis in NSCLC. The difference between findings may be due to different choices of treatment and different components of data.

BRCA1 is implicated in NER, which was discussed in the part of ERCC1, it also associates with double-strand break repair and mismatch repair, indicating its crucial role in DNA repair [25]. It has been indicated that BRCA1 presents different sensitivity to different chemotherapy agent in vitro study. The negative expression of BRCA1 results in high sensitivity to cisplatin, whereas its positive expression increases sensitivity to antimicrotubule agents [26]. In clinical research, it was found that patients whose tumors had BRCA1 expression would have significantly poorer survival and should be candidates for adjuvant chemotherapy [27]. Median survival was 11 months for 38 patients with low BRCA1, treated with cisplatin plus gemcitabine; 9 months for 40 patients with intermediate BRCA1, treated with cisplatin plus docetaxel; and 11 months for 33 patients with high BRCA1, treated with docetaxel alone. Two-year survival was 41.2%, 15.6% and 0%, respectively, which had manifested the potential predictive role of BRCA1 in a recent non-randomized phase II clinical trial [28]. Our findings indicate that BRCA1 expression might correlate with OS and platinum treatment based OS, however, there was no statistical significance (*P *= 0.052 vs. *P *= 0.073). Nevertheless, the results tend to migrate to statistical significant directions accompanied extension of follow-up time and expansion of sample size.

In addition, as the gene sensitive to cisplatin or other DNA damaging agents, expression of ERCC1 is closely related to BRCA1, no matter in breast cancer or in NSCLC [29,30]. But there is not much more studies indicate correlations between BAG-1. Our findings demonstrate a strong correlation between ERCC1 and BAG-1. Therefore, it is plausible that patients with the expression of ERCC1 and BAG-1 present a poor prognosis and the lack of its expression would receive more benefit from non platinum based chemotherapy.

As one of the targets of gemcitabine, RRM1 also have roles in DNA repair systems like ERCC1 and BRCA1. It encodes the regulatory subunit of ribonucleotide reduction of ribonucleoside diphosphates to the corresponding deoxyribonucleotides [31]. In earlier study, it suggested continuous exposure of lung cancer cell lines to increasing amounts of gemcitabine resulted in increased expression of RRM1 [32]. In addition, another research showed reduced RRM1 expression increased sensitivity to gemcitabine in lung cancer cell lines, and found RRM1 expression in tumor is a major predictor of disease response to gemcitabine chemotherapy during a prospective phaseII clinical trial with NSCLC [8]. TUBB3 is investigated and recognized as a role in resistance to antitubulin agents. The report shows TUBB3 is expressed in high levels in lung cancer cell lines, and by using RNAi technology, it was found that TUBB3 mediates sensitivity to paclitaxel in NSCLC cells, and high levels of TUBB3 expression are associated with paclitaxel and docetaxel resistance in vitro [11,33,34]. Our result showed that TUBB3 was more frequently observed in stage I + II than in stage III + IV patients (*P *= 0.004). But Recent data suggested expression of TUBB3 was related to advanced stage NSCLC [35]. In this study, no correlation of chemotherapy between RRM1 and TUBB3, or the survival of the patients was found. It might be caused by the limitation of different cycles of adjuvant chemotherapy taken by patients and other interferences like number of samples and only one clinical center involved in our study.

## Conclusions

In summary, to better overcome the problems related to drug resistance and to improve the clinical outcome of advanced NSCLC patients, relationship between drug resistance caused by gene expression and prognosis of patients received adjuvant chemotherapy must be investigated. Our findings indicate ERCC1 and BAG-1 are prognostic factors for progression-free and overall survival, and may be predictive biomarkers for platinum based chemotherapy in NSCLC patients. Accompanied by enlargement of sample size, BRCA1 might also be an indicator the above-mentioned. Although the approach of RT-PCR has a better feasibility and repeatability, and we have quality control of the laboratory. It remain has many factors influence the experimentation to cause the false positive results. Moreover, 85 patients were certainly few and follow-up time was short to be able to conclude firmly on any of the findings in our study, particularly using multivariate analysis. However, because of patients with negative expression of these genes indeed receive more benefit from platinum based chemotherapy in our study, the combined detection of the mRNA expression of these genes might better individualize the efficacy of chemotherapy and improve survival in this common and vital cancer.

## Funding

This research was supported by Guangxi Scientific research and technology development projects (Grant No. 10124001A-44)

## Competing interests

The authors declare that they have no competing interests.

## Authors' contributions

XFL: RT-PCR operations, statistical analysis, collection of patients' information, manuscript drafting. MWC: Research planning, statistical analysis, manuscript drafting. LX: Research planning, surgery and maintenance of patients' database. LD: RT-PCR operations. GYM: RT-PCR operations, data sorting and processing. MHL: Patients' data sorting and processing. All authors read and approved the final manuscript.
